# A blended face-to-face and smartphone intervention for suicide prevention in the construction industry: protocol for a randomized controlled trial with MATES in Construction

**DOI:** 10.1186/s12888-019-2142-3

**Published:** 2019-05-14

**Authors:** A. Milner, T. L. King, A. J. Scovelle, P. J. Batterham, B. Kelly, A. D. LaMontagne, S. B. Harvey, J. Gullestrup, C. Lockwood

**Affiliations:** 1 Centre for Health Equity, Melbourne School of Population and Global Health, the University of Melbourne, Melbourne, Victoria 3010 Australia; 2 Centre for Mental Health Research, Research School of Population Health, The Australian National University, Canberra, ACT 2601 Australia; 3 School of Medicine and Public Health, University of Newcastle, Newcastle, New South Wales 2308 Australia; 4 Centre for Population Health Research, School of Health & Social Development, Deakin University, Geelong, Victoria Australia; 5 Black Dog Institute, Faculty of Medicine, University of New South Wales, Sydney, Australia; 6 MATES in Construction, Spring Hill, Queensland 4004 Australia

**Keywords:** Mental health, Help-seeking, Help-offering, Suicide prevention, Men, Employment, Workplace

## Abstract

**Background:**

Construction workers are at elevated risk of suicide. MATES in Construction (MATES) is one of the few suicide prevention programs that explicitly address this problem. The MATES program includes an integrated system of services that supports prevention, early intervention and recovery (i.e., primary, secondary and tertiary prevention) for mental health problems among construction workers. In this protocol, we describe a proposed evaluation of *MATESmobile*, an electronic platform which will be accessed by workers who have undergone MATES training.

**Methods/design:**

In this protocol, we describe a Randomised Controlled Trial (RCT) which seeks to assess whether *MATESmobile* results in improved literacy regarding suicide prevention, and improved help-seeking and help-offering attitudes among those who have attended MATES training. Secondary outcomes include changes in suicide ideation, suicide attempt and psychological distress. Workers will be recruited prior to MATES face-to-face training. In total, 295 workers will be randomly assigned to the intervention condition (*MATESmobile* + face-to-face training) and 295 will be randomly allocated to the control (face-to-face training). The intervention will run for 8 weeks. Assessments will be run immediately post intervention, and at 3, 6, and 12 months

**Discussion:**

*MATESmobile* offers the potential to reinforce and enhance the effects of face-to-face training, resulting in greater skills and knowledge in suicide prevention, as well as a reduction in suicidality and distress.

**Trial registration:**

This trial is registered with the Australian New Zealand Clinical Trials Registry (ACTRN12619000625178; 26 April 2019).

## Background

Elevated rates of suicide among construction workers have been identified in many countries [1], including Australia [2]. Such elevated rates among construction workers may reflect a range of factors connected to employment in a highly-male dominated workforce, including excessive alcohol consumption, relationship problems, and lack of help-seeking [3, 4]. Aside from this, workers in construction also face a number of adverse psychosocial working conditions [5], including low job control and high psychological demands (the combination of which produces job strain) [6], low social support [7], and work-family conflict (when role pressures from the work and family domains are mutually incompatible) [8]. There are constant shifts in the landscape and organisation of construction work, as tasks, colleagues and supervisors change from project to project and job to job, contributing to feelings of job insecurity [9]. Adding to this instability is the fact that many workers are forced to meet short deadlines and experience periods of unemployment between projects [9]. The lowest skilled groups may be at most risk, especially during times of economic recession [10].

Mates in Construction (MATES) is a charity established in 2008 by the Building Employees Redundancy Trust to prevent suicide in the construction industry. MATES is an industry-based, multimodal workplace-focused charity, delivered at construction sites or company offices. Significant commitment from building site management to the program has been crucial — both to communicate organisational investment in addressing the issue of suicide, and also allow training to be completed during work hours. MATES is a multi-component prevention and early intervention program, consistent with the Australian National Suicide Prevention Strategy (‘Living Is For Everyone’ or ‘LIFE’) [11]. MATES operates over four states and, to date, training has been delivered to over 135,000 workers. The core component of the MATES training is the General Awareness Training (GAT), a 45-min face-to-face awareness session provided to all construction workers on site. GAT promotes awareness of and risk factors for suicidality. It aims to reduce stigma and encourage help-seeking and help-offering behaviour, and presents suicide as preventable. “Help offering” refers to workers offering active support to co-workers who display any suicide warning signs. Workers completing GAT are provided with a white sticker to wear on their hard hat identifying them as ‘GAT-trained’. For a site to be designated ‘compliant’, all workers on that worksite must receive GAT, with an 80% training level maintained despite staff turnover. GAT is also a component of a training program specifically for apprentices. Other components of MATES that support GAT include:

- “Connector training” for volunteer “gatekeepers”: Connectors help an at-risk worker access help via a trained worker, MATES Field Officer, Case Manager or other local community supports.
- Suicide First Aid: in which Applied Suicide Intervention Skills Training (ASIST) trained workers provide suicide first aid interventions for at-risk workers identified by Connectors.
- Field Officers: Field Officers are employed directly by MATES. Their job is to increase awareness, recruit new construction sites, and provide ongoing support to MATES sites through fortnightly site visits, establishing and maintaining relationships with workers on-site, and debriefing Connectors.
- Case managers: Suitably qualified case managers are employed to assist troubled workers with a plan to effectively address their issue(s). This could include connecting workers with such services as their EAP, financial counselling, drug and alcohol services, grief counselling, or family and relationship counselling.

Thus, MATES provides an integrated health service model aimed at prevention, early intervention, and recovery (i.e., primary, secondary and tertiary prevention).

Since its inception, MATES has been committed to evaluation and continual program improvement. A recent evaluation [12] compared age-standardised suicide rates among the construction industry to other employed males. There was evidence of a decline in suicide rates among construction workers employed in the state of Queensland (where there has been a high uptake of MATES) compared to other employed males. In 2011, another evaluation [3] assessed process related outcomes, for example, how prepared participants felt to deal with someone who was suicidal, and attitudes towards suicide. Compared to 355 workers at two sites who had not completed GAT, those who received the training showed greater improvements in knowledge about suicide.

Several other uncontrolled evaluations have also suggested that MATES is an acceptable and appropriate intervention for workers in the construction industry [13–16]. The main limitations of these evaluations are that they only assessed knowledge immediately before and after training, and therefore could not provide information on whether MATES resulted in greater retention of knowledge in the long term. Thus, this finding may reflect transient “training effects” – where an individual retains knowledge immediately post training but loses it over time. Another problem is that these evaluations were conducted in one state only, and the findings may therefore not be generalisable to other states. Lastly, these evaluations did not assess the extent to which training reduced suicide attempts and thoughts of suicide among construction workers. Thus, several questions remain about the extent to which those who take part in the program experience individual changes in relevant outcomes proximal to suicide death, such as suicide ideation, self-harm or suicide attempt, and psychological distress. Further, and most specific to this project, questions remain about the longer-term impacts of the program, and whether it would achieve better outcomes if strengthened and reinforced by additional elements.

To fill the gaps in knowledge noted above, MATES has recently developed *MATESmobile*, an electronic platform which was designed to follow MATES face-to-face GAT training. *MATESmobile* seeks to enhance and sustain the effectiveness of MATES through a smartphone approach, which we will evaluate in relation to a wider range of outcomes than assessed previously. *MATESmobile* will focus on two main elements: 1) reinforcing face-to-face training messages over time, and; 2) enabling links to mental health support should people need it. The use of a smartphone approach to deliver this program is feasible and appropriate considering the high uptake of smart phones the construction industry, where they are considered a ‘tool of trade’.

In this paper, we describe the randomised controlled trial to test the efficacy of *MATESmobile.* The evaluation seeks to evaluate the potential gains of complementing MATES face-to-face training with *MATESmobile*. *MATESmobile* will be implemented by MATES, with support from the research team named in this project. This trial is registered with the Australian New Zealand Clinical Trials Registry (ACTRN12619000625178; 26 April 2019).

## Methods

### Design

This is a two-arm individual participant randomised controlled design, following SPIRIT guidelines [17, 18].

The day-to-day management and administration of *MATESmobile* (including recruitment, roll out of intervention, and dissemination and collection of survey information) will be the responsibility of MATES.

### Program logic

**Fig. 1:**
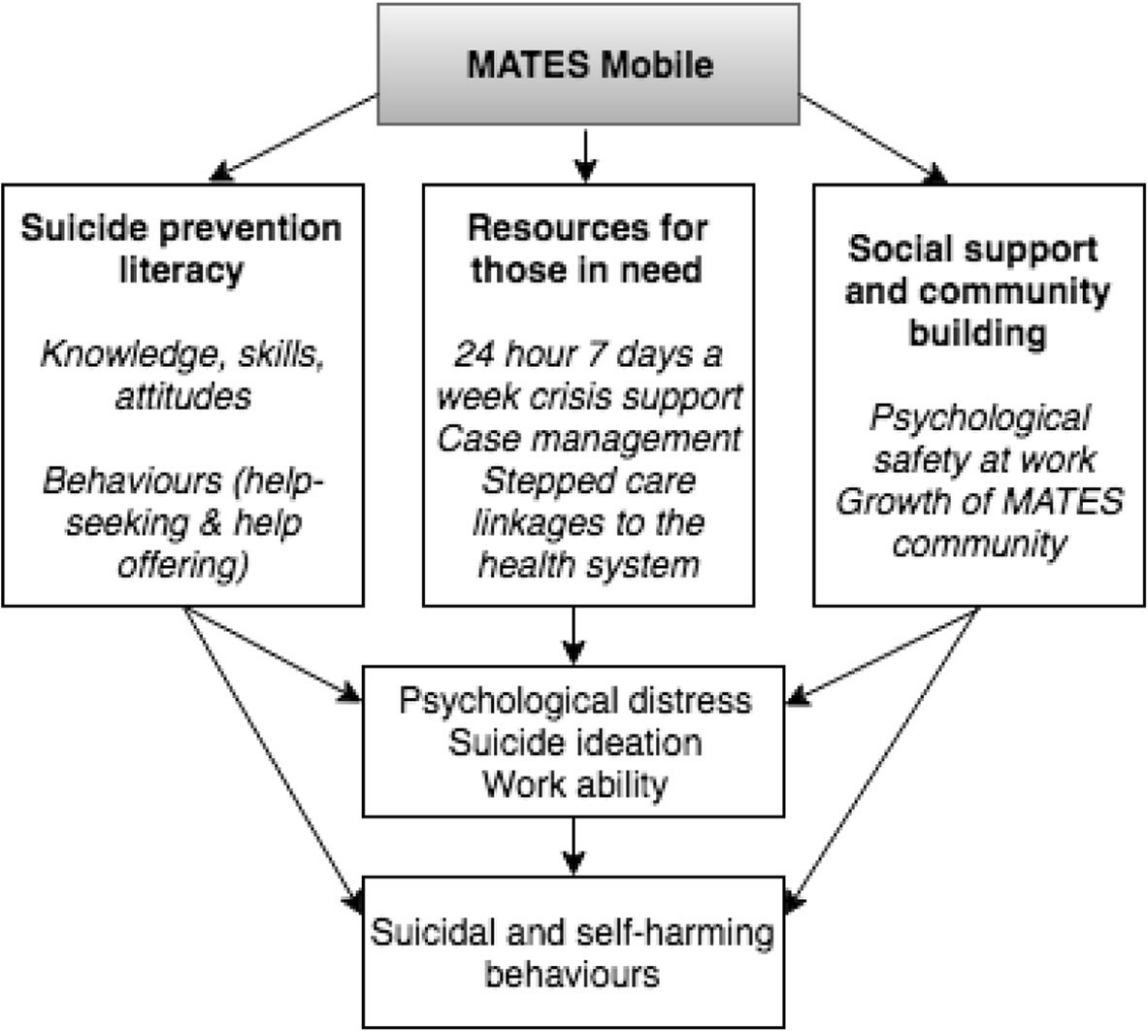
Program logic for MATESmobile

The program logic of *MATESmobile* can be seen in Fig. 1. *MATESmobile* will have ongoing contact with individual participants, approximately once per fortnight over an 8-week period.

This ongoing contact will reinforce messages from MATES, aiming to increase knowledge and skills in suicide prevention literacy, as well as more helpful attitudes about the extent to which suicide is preventable. The reinforcement of messages will also flow through to impact help offering (e.g., the message “MATES help other MATES”) and also help-seeking from either formal (e.g., doctors, mental health professionals, case management) or informal sources (e.g., peer support from co-workers, friends and family). At the same time, the *MATESmobile* platform will provide informational resources for those in need or at risk of suicide, including how to access to case management and other health services. It also seeks to encourage social support and community around the MATES program, leading to improvements in psychosocial safety at work. Collectively, all these aspects of the program are hypothesized to lead to improvements in primary and secondary outcomes.

### Field testing

The content of *MATESMobile* was designed by the founding organisation of the MATES program, including the National and State Chief Executive Officers and field staff. Staff employed at MATES were consulted to design program materials that were relevant and appropriately framed for the industry. This included input into the frequency and timing of content delivery, as well as the attractiveness, clarity, and usefulness of the content.

### Study setting

The study will be set in construction sites across Australia. These sites will include those managed by the Industry Partners involved in the study (who are Australia’s leading construction companies), and/or sites where MATES is operational.

### Eligibility criteria

Participants need to be recruited from a site where MATES is operational. Workers attending GAT will be considered eligible for the study.

### Study objectives and outcomes

The primary objective of the evaluation is to assess whether *MATESmobile*: a) improves suicide prevention literacy, as measured by the GAT suicide awareness questionnaire [3]; and b) improves help-seeking and help-offering attitudes, as measured by the General Help-Seeking Behaviour Questionnaire [19], and the GAT help-seeking and help-offering items. Secondary aims are to assess whether *MATESmobile* results in greater reduction in suicide ideation as measured by the 4-item Suicidal Behavior Questionnaire-Revised (SBQ-R) [20] as well as psychological distress, as measured through the Kessler-6 [21]. Changes in suicide attempt will be measured as an exploratory outcome (anticipating a potential low incidence of this outcome).

### Interventions

#### Training of field officers

There will be a standardised recruitment procedure across different construction sites. The research team will ensure that MATES field staff are well informed about both the program, and how to explain it to potential participants. The MATES Project Manager will train field officers about how to introduce and recruit participants to the study.

#### Intervention condition

After completion of standard GAT face-to-face training, those allocated to the *MATESmobile* program will be contacted via smartphone application every two weeks for 8 weeks with information about how to access the program and reminders. We will conduct baseline assessment (a longer survey) prior to the 8 week intervention, followed by smaller assessments at 3, 6 and 12 months.

#### Control condition

Those in the control arm will complete a baseline survey, GAT training, and additional surveys at 3, 6 and 12 months following baseline.

### Recruitment of participants

Information about *MATESmobile* will be communicated at the start of GAT by MATES field officers, prior to the delivery of the GAT content. Field officers will provide information on the project including plain language statements and the procedure for providing consent. Those who are interested in the project will be asked to complete a written baseline survey, a written consent form and provide their mobile phone number. These will be placed in a sealed cardboard box during GAT, and will be returned to the project manager at MATES. The field officers will assure participants that this identifying information will not be provided to employers or released to any other party, and that results from the study will only be available in aggregate form, with no identifying information revealed. No participants have been recruited to the trial at this time.

### Ethics and provision of care

All enrolled participants will be required to provide written consent to being involved in *MATESmobile.* If any person is identified as being at risk of suicide at the time of GAT or at a later time (identified through the survey items, e.g., the SBQ-R [20]), they will be offered connection to MATES case management and other suicide prevention help-lines, irrespective of which condition they are allocated to.

### Sample size and power calculations

Previous work on examining suicide prevention literacy in construction workers using MATES [22] found a mean difference of 0.429 on a scale of one to five in beliefs about suicide warning signs at post-intervention. Based on this, a minimum of 295 participants per group will be required to detect this difference at the nominal 0.05 significance level with 90% power. A previous study has shown that 13% of the working population report suicide ideation [23]. We believe it is realistic to expect a change from 13 to 8% prevalence following the trial. Power calculations indicate we will need 267 persons per group to detect this difference at the nominal 0.05 significance level with 90% power. Assuming 30% drop-out, we therefore aimed to recruit a total of 844 participants over a three-month period.

### Data collection

Participants in both conditions will complete online surveys (following the written baseline survey), which will be delivered to their smart phone via “alerts”. The “alerts” will re-direct respondents to an online survey. The surveys will be confidential, but not anonymous, as we will need to link baseline to follow up surveys, which will be done using mobile telephone numbers. Identifying information will also enable personalised re-contacts for non-response (which may be conducted via email and/or text messaging). The app will be available on Android, iOS and Windows mobile systems.

### Allocation sequence

Allocation concealment will be achieved by generating a set of computerised random numbers which will be assigned to participants. We will randomise in blocks of 50 participants. Hence, once 50 persons have returned their consent form, they will be allocated a random number. Once a block of 50 participants is obtained, the random numbers will be sent to a statistician external to the trial for allocation (discussed below).

### Blinding

Those involved in the design and evaluation of the trial will be blinded to participants’ allocation status. It is not possible to blind participants to the conditions they are allocated to. Nor is it possible to blind the project manager, who will be based at MATES.

### Implementation

The project manager will enrol participants. The statistician will generate the allocation sequence and assign participants to intervention or control conditions following randomisation.

### Randomisation

Participation numbers will be provided to a statistician external to the trial who will randomise participants into either the *MATESmobile* condition or control condition (MATES face-to-face only). The statistician will generate the allocation sequence for randomisation using a computer-based randomisation algorithm. The statistician will not reveal which of the participants are allocated to the intervention or control conditions to those involved in the design and evaluation process.

### Measures

Socio-demographic data (e.g., age, sex, educational level) will be collected using the standard survey questions as collected by the Australian Bureau of Statistics [24]. Suicide prevention literacy will be assessed with the GAT suicide awareness questionnaire, which asks about suicide knowledge, attitudes, and behaviours [3]; help-seeking and help-offering will be assessed with the General Help-Seeking Questionnaire [20] and the GAT help-seeking and help-offering items. Psychological distress will be measured using the Kessler-6 instrument [21]; psychosocial risks in the workplace will be assessed using the Psychosocial Safety Climate (PSC-12) [25]; and suicidal ideation and behaviours will be assessed using the revised version of the Suicidal Behavior Questionnaire-Revised [20].

### Process evaluation

User engagement metrics will be recorded via the app. Information such as: i) number of times engaged with the app; ii) time spent using the app per session; and iii) number of pages viewed per session, will be recorded. Additionally, participants will be asked to respond to survey questions regarding their experience of the app, such as: i) what did you like about *MATESmobile*?; ii) what do you think could be improved about *MATESmobile*?; iii) would you recommend *MATESmobile* to a colleague/friend? These questions will be sent to participants following their completion of the final survey.

### Data management and monitoring

Data will be obtained from: a) written responses to a baseline paper survey; b) electronic responses to post-baseline surveys. Responses to the baseline data survey will be entered into an electronic file (e.g., Microsoft excel) by the Project Manager based at MATES, who will enter this data twice to ensure accuracy. The paper files will be stored in a locked cabinet only accessible by the Project Manager. The Project Manager will assign a participant identifier (based on the mobile phone number that the participant provides). Once participants are allocated to an intervention or control condition, the research team will be sent the baseline data file electronically via a secure server.

The remaining surveys will be completed electronically via smartphones, and captured using an electronic survey platform. The Project Manager will have access to this survey platform and will monitor responses to the survey. The Project Manager will be able to link baseline survey information to follow up surveys using the mobile phone number provided by the participants. There will be an inbuilt prompt within *MATESmobile* which will be sent to participants to remind them to complete surveys. This will be sent 2 days after the survey is initially sent. Following this, two other messages will be sent to prompt participants to complete the survey on the third day and then again on the fourth day. On the seventh day, the Project Manager will phone participants and ask them to complete the survey. A data monitoring committee was not required by the Ethics Committee for this trial as the risk of harm to participants is negligible.

### Analysis

Analyses of continuous outcomes will be undertaken on an intent-to-treat basis, including all participants randomised, regardless of treatment actually received or withdrawal from the study. Mixed-model repeated measures (MMRM) analyses will be used because of the ability of this approach to include participants with missing data without using correction biased techniques, such as last observation carried forward [26]. This approach can also accommodate and assess the strength and significance of clustering effects, enabling us to model cohort effects (including training time and location) using random effects within the MMRM models. For binary outcomes (prevalence of suicidal ideation) and skewed outcomes (severity of suicidal thoughts and behaviours), a comparable modelling approach will be used that accounts for binary and ordinal outcomes. Process evaluation will be analysed using descriptive statistics; for example, the average amount of time spent using the app during each session will be assessed. Responses to the process survey, sent to participants following completion of the trial, will be examined. The mean and frequency of quantitative responses will be calculated, and content analysis of qualitative responses will occur. Quantitative analyses will be undertaken in Stata for Windows, version 15 [27].

### Ethics and dissemination

Ethical approval has been granted by the University of Melbourne (ID: 1852200). Important protocol modifications will be communicated to the research team, the ethics committee and participants. As mentioned above, written informed consent will be obtained from participants at the time they complete the baseline survey. The authors declare no conflicts of interest. A plain language report on the results of the trial will be publicly available on the MATES website following completion of the evaluation study. Outcomes of the study will also be communicated in academic journal articles and conference presentations. Authors on all publications will be required to meet the academic authorship guidelines.

## Discussion

Suicide among construction workers continues to be unacceptably high in Australia, as in many areas of the world. MATES is one of the few workplace suicide prevention programs attempting to tackle the high burden of suicide in this occupational group. Importantly, it is among the few workplace suicide prevention program for which there is any evidence of effectiveness [28]. However, there are still much to be learned about MATES, including whether training effects persist over time, and whether the program has effects on proximal risk factors for suicide, such as suicide ideation, suicide attempts and psychological distress. We hope that the *MATESmobile* trial will begin to fill some of these gaps in current knowledge, and provide evidence for the effectiveness of a new, highly accessible smartphone app that can be rapidly disseminated throughout the construction industry.

Online and electronic interventions have been shown to be effective in reducing stigmatising attitudes towards depression [29], enhancing knowledge about evidence-based treatment, promoting help-seeking from family and friends [30], and improving knowledge about symptoms of depression and anxiety [31]. However, their effectiveness in enhancing knowledge about suicide prevention, suicide ideation, and self-harm is mixed [32]. Researchers acknowledge that online interventions are most effective when supported by strategies that: allow users to tailor activities to their context; reduce attrition; and promote ongoing use [33]. This presents a clear rationale for an approach that makes use of both online and face-to-face mechanisms (i.e. a blended approach). In this study, we will assess the potential gains that can be made by supplementing a multi-level workplace suicide prevention program with smartphone technology, which will allow MATES to keep in contact with participants over time and as they move from site to site.

Aside from testing the effectiveness of *MATESmobile*, the Project offers the opportunity for considerable capacity-building between health service provider MATES, and the Partner Organisations who are supporting the project; all of whom view *MATESmobile* as a platform for extending their activities to other aspects of workplace health and wellbeing. *MATESmobile* has a multitude of potential future applications and uses, including being a way of connecting and supporting men and women in the construction industry long term, and thereby creating a community of advocates for suicide prevention.
